# Euthanasia in advanced dementia; the view of the general practitioners in the Netherlands on a vignette case along the juridical and ethical dispute

**DOI:** 10.1186/s12875-021-01580-z

**Published:** 2021-11-18

**Authors:** Jaap Schuurmans, Chantalle Crol, Boudewijn Chabot, Marcel Olde Rikkert, Yvonne Engels

**Affiliations:** 1 General practice Ottenhoff, B. Ottenhoffstraat 18, 6561 CM Groesbeek, The Netherlands; 2 Radboud University Medical Center, Postbox 9101, 6500 HB Nijmegen, The Netherlands

**Keywords:** Euthanasia, Dementia, General practitioners, Support, Burden, Jurisdiction, Human rights, Ethical implications

## Abstract

**Background:**

In the Netherlands, euthanasia has been regulated by law since 2002. In the past decade, a growing number of persons with dementia requested for euthanasia, and more requests were granted. A euthanasia request from a patient with advanced dementia (PWAD) can have a major impact on a general practitioner (GP). We aimed to get insights in the views of Dutch GPs on euthanasia concerning this patient group.

**Methods:**

A postal survey was sent to 894 Dutch GPs. Questions were asked about a case vignette about a PWAD who was not able to confirm previous wishes anymore. Quantitative data were analyzed with descriptive statistics.

**Results:**

Of the 894 GPs approached, 422 (47.3%) completed the survey. One hundred seventy-eight GPs (42.2%) did not agree with the statement that an Advance Euthanasia Directive (AED) can replace an oral request if communication with the patient concerned has become impossible. About half of the respondents (209; 49.5%) did not agree that the family can initiate a euthanasia trajectory, 95 GPs (22.5%) would accept such a family initiative and 110 GPs (26.1%) would under certain conditions.

**Discussion:**

In case of a PWAD, when confirming previous wishes is not possible anymore, about half of the Dutch GPs would not accept an AED to replace verbal or non-verbal conformation nor consider performing euthanasia; a minority would. Our study shows that, probably due to the public debate and changed professional guidelines, conflicting views have arisen among Dutch GPs about interpretation of moral, ethical values considering AED and PWADs.

**Supplementary Information:**

The online version contains supplementary material available at 10.1186/s12875-021-01580-z.

## Background

Death according to personal preference and in a manner that resonates with the person’s individuality is increasingly considered as an important element of ‘a good death’ in modern Western culture [1]. Consequently, an increasing number of countries legalizes euthanasia. Although there are more countries where euthanasia also can be provided to persons with dementia (PWDs), only in the Netherlands an advance euthanasia directive (AED) can replace a verbal request for euthanasia in a later stage of dementia, if all other obligatory criteria are met [2–4].

Initially, like in other countries, the large majority of euthanasia requests and acts concerned terminal patients with cancer [5]. However, during the last decade, the Dutch number of euthanasia cases in persons with dementia (PWDs) has increased from 25 (of 3136 cases in total) in 2010 to 162 cases (of 6361 in total) in 2019 [6]. Indeed, Dutch society, influenced by the growing media and political attention, considers dementia as a debilitating and degrading disease and by many as synonymous with unbearable suffering [7–9]. As most PWDs, especially in the early stages of the disease, live at their own home [10], particularly GPs are confronted with euthanasia requests of PWDs [5]; a growing number of people in the Netherlands discuss and share an AED with their GP [11].

Recent studies showed that dealing with AEDs, euthanasia requests and procedures often have become a burden for GPs; they experience pressure from relatives, have problems with judging mental capacity of PWDs, and the Dutch society’s stigmatization of dementia [12–14]. Not having the same expectations as relatives, as well as disagreeing with relatives about AEDs, (un)willingness to perform euthanasia and if, its timing of euthanasia, contribute to this burden [15–17]. (Re)discovering the right balance between the physician’s professional responsibility, and in such cases, the patient’s and relatives’ autonomy has been recommended [15].

Until 2015, the Royal Dutch Medical Association (KNMG) directed that, on medical-ethical grounds, it was necessary that the patient confirmed his or her actual death wish, verbally or non-verbally, when receiving euthanasia, regardless of having an AED. In 2015, the KNMG published its latest guideline, following more liberal possibilities than given by law, in which an AED was not required as stipulates in section 2.2 (Table 1) [18]. This can be considered a baseline shift, responding to the society expectations to provide maximum juridical space for PWDs.

**Table 1:** The requirements of due care in Dutch law as stipulated in the Article 2 of The Termination of Life on Request and Assisted Suicide Act

Under the law, the definition of euthanasia applies when a physician ends the life of a patient at his express request due to unbearable and lasting suffering. Euthanasia means that the physician administers a lethal substance to the patient. In the case of assisted suicide, the physician supplies a lethal substance that the patient takes in the physician’s presence. The physician must: a. Be satisfied that the patient’s request is voluntary and well considered. b. Be satisfied that the patient’s suffering is unbearable, with no prospect of improvement. c. Have informed the patient about his situation and his prognosis. d. Have come to the conclusion, together with the patient, that there is no reasonable alternative in the patient’s situation. e. Have consulted at least one other, independent physician, who must see the patient and give a written opinion on whether the due care criteria set out in (a) to (d) have been fulfilled. f. Have exercised due medical care and attention in terminating the patient’s life or assisting in his suicide. The Act stipulates in section 2.2 that a patient aged 16 or over who is decisional competent may draw up an advance directive, setting out a request for euthanasia. If at some point the patient is no longer capable of expressing his will, the physician may accept the advance directive as a request pursuant to section 2 (1)(a) of the Act.1 2 The advance directive thus has the same status as an oral request for euthanasia	

Recently, a Dutch euthanasia case concerning a woman with advanced dementia was tested against criminal law, to acquire jurisprudence, thereby seeking formal ground for this legal option. This even increased GPs’ concerns around euthanasia in PWDs [19]. The Supreme Court in The Hague determined that the woman with advanced dementia in question who was given euthanasia based on her AED without actual confirmation of her request, legally and professionally received sound care in line with the amendment of the law. This case, in which the physician had been accused of murder, was dismissed [20, 21].

Although this case concerned an elderly care physician working in a nursing home, this first ever euthanasia court case was considered as threatening in primary care across the Netherlands. As a result GPs typically are confronted with euthanasia requests and AEDs, and carry out 85% of all euthanasia procedures [22]. Clearly, there are professional and legal challenges and ethical concerns that GPs face when dealing with euthanasia requests and AEDs from PWDs. Therefore we aimed to answer the following research questions: What are the views of Dutch GPs on euthanasia concerning patients with advanced dementia (PWAD)?

## Methods

### Study design and participants

Between January and March 2019, we performed a quantitative postal survey. The addresses of a representative sample of 894 Dutch GPs were received from the Dutch institute for healthcare research (NIVEL), containing the majority of Dutch GPs. These GPs gave their consent for sharing their postal addresses for research purposes. GPs, with or without experience with euthanasia requests or procedures in general or with PWDs specifically, were invited to take part, regardless of their opinion about euthanasia. Exclusion criteria were being retired GPs or not working as a GP anymore.

### Survey

Since no validated questionnaire to answer our research question was available, and no comparable study had been performed before, a survey was developed (Additional file 1). Based on a literature search, a qualitative interview study [12] and two expert meetings [23], a concept survey was composed. Face validity of the survey was achieved through pilot testing by six GPs, an ethicist, a journalist, a geriatrician and an elderly psychiatrist, and adapted where necessary.

The survey took 15 min to complete.

The survey started with questions characterizing personal and clinical practice context. Next, questions on AED and euthanasia requests from PWD followed which have been published elsewhere [13]. Here, we focus on the questions that concern the view of GPs on euthanasia in PWADs. These concern:

1. A 5 point Likert scale (totally disagree-totally agree) on having problems with judging the specific criterion of unbearable suffering in a competent and in an incompetent patient.
2. After presentation of a case vignette, which was inspired on the recent juridical case that concerned euthanasia for a person with advanced dementia (Table 2), the respondents were asked their judgement whether or not the GP acted correctly. (yes or no) Next some additional questions (yes, no, maybe) were asked:
  - Can an AED replace an oral request if communication with a PWAD is not possible anymore?
  - Can the family initiate a euthanasia procedure and represent the interests of a PWAD?
  - Is it allowed that a PWAD is sedated before euthanasia is performed?

**Table 2:** Case Vignette

*Mr. Smit is 70 years old and is indisputably diagnosed with dementia by a geriatrician. He does not recognize his wife and children anymore, refuses to eat, and increasingly isolates himself. Discussing his treatment is not possible anymore. Ten months ago, still being competent, he composed an advance euthanasia directive (AED), in which he declared that he would opt for euthanasia when suffering from dementia.* *His family is now asking for performing this, given the patient’s AED and his unbearable suffering with no prospect of improvement. The general practitioner considers the patient incompetent, can imagine that the patient is unbearably suffering and is convinced that the patient’s AED can replace an oral request. The consulted SCEN physician*^*a*^ *and elderly care physician confirmed this and approved euthanasia. A sedative was orally administered to prevent possible unpredictable behavior, agitation and startle reactions at which the patient might walk away, after which the GP performed the euthanasia. After having received the written report of the euthanasia procedure from the GP, the regional review committee invites him to give an explanation of his actions.*	

^a^SCEN; support and consultation on euthanasia in the Netherlands. SCEN physicians are available for support, information and formal consultation around euthanasia

In an open text box, the GPs could explain their answers.

### Procedure

**Fig. 1:**
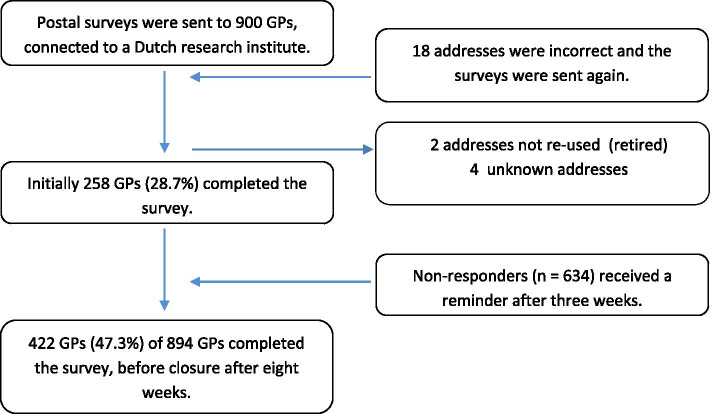
Study flow diagram

A code list was generated for the unique codes of the surveys and names of the GPs. The survey, for each GP with a unique code, an information letter and a self-addressed return envelope were sent in January 2019 to the GPs by regular mail. Non-responders received a reminder 3 weeks later. The study flow diagram is shown in Fig. 1.

Data of the completed surveys were entered in Castor EDC, a cloud-based clinical data management platform, and after closure of the database transported to SPSS.

### Statistical analysis

All data were analyzed using SPSS software version 25. Frequencies with percentages and means with standard deviations (SDs) were used as descriptive variables.

## Results

### Recruitment

Of the 894 included GPs, 423 (47.3%) completed the survey. The study flow diagram, describing the procedure and response rate initially and after a reminder is shown in the Fig. 1.

### Characteristics of the GPs’

There was an equal division between males and females, and the majority of the GPs worked as a regular in a general practice. The mean age was 48 years with a mean of 17 years’ experience (Table 3).

**Table 3:** GPs’ characteristics (*N* = 423)

Characteristics.	*N* (%)^a^	Mean (SD)
Age in years		48.1 (9.8)
Experience as GP in years		16.5 (9.4)
Gender		
Male	207 (49.2)	
Female	214 (50.8)	
Kind of GP		
Regular	390 (92.6)	
Locum	30 (7.1)	

^a^ Number of missing variables among 2-17 GPs

### Judging the due criteria

Of the responding GPs, 348 (82.4%) (totally) agreed that it is difficult to judge the due care criterion ‘unbearable suffering with no future improvement’ of an *incompetent* person with dementia (PWAD).

When it concerns a *competent* PWD who considers his perspective of future suffering as unbearable, 247 GPs (58.6%) (totally) disagreed that in such a case the due care criteria are met.

### Case vignette

On the question if the GP in the case vignette acted correctly, 178 (42.2%) answered with ‘yes’ and 210 (49.8%) with ‘no’ (Table 4). Seventy-six GPs (18%) agreed that an AED can replace an oral request if communication with the concerned patient has become impossible, 178 did not agree (42.2%) and 158 (37.4%) answered ‘maybe’.

**Table 4:** Case Vignette and personal view

		Number of GPs that responded to the question	Yes n (%)	No n (%)	Maybe N (%)
25a	Do you judge the way of acting of this GP as correct?	388	178 (45.9)	210 (54.1)	–
25b	An AED can replace an oral request if communication with the concerned patient is impossible.	412	76 (18.0)	178 (42.2)	158 (37.4)
25c	The family can initiate the start of a euthanasia procedure representing the interests of the concerned patient.	414	95(22.5)	209 (49.5)	110 (26.1)
25d	A sedative medicine prior performing euthanasia to the concerned patient is allowed.	396	181 (42.9)	126 (29.9)	89 (21.1)

When stating that the family can initiate the start of a euthanasia procedure representing the interests of the concerned patient (in case this patient is not able anymore to confirm such a previously documented wish), 95 (22.5%) GPs agreed, while 209 (49.5%) disagreed, and 110 (26.1) said ‘maybe’. GPs added in the open text box explanations like: ‘this is only an option if I know the family and patient well’; ‘if the patient has authorized this family member earlier when he or she was still mentally capable’, or ‘if the family participated in earlier conversations about euthanasia and if there is no pressure from relatives or conflict of interests’.

Administration of sedative medication prior the euthanasia performance was agreed by 181 GPs (42.9%) and disagreed by 126 (29.9%) GPs, while 89 GPs (21.1%) found this only acceptable if, as mentioned in the open text boxes, the patient is restless and if it was discussed and documented in the AED.

## Discussion

When it concerns euthanasia in PWAD, the vast majority of Dutch GPs experienced difficulties to adequately judge the for euthanasia obligatory criterion of unbearable suffering with no prospective improvement. After having read a case vignette which was based on the recent Dutch juridical case around euthanasia in a PWAD, only one out of five GPs agreed that an AED can replace an oral request in PWADs and that the family can initiate the euthanasia procedure. One-third of the GPs in our study had objections against sedation prior to performing euthanasia, even though this is allowed according to the currently being updated Dutch guideline on performance of euthanasia [24]. One out of five to one out of three GPs could not answer these questions with a clear yes or no.

In our fictive case vignette, the several elements of the first legal case where the physician was accused of murder, were integrated. At the moment the GPs completed the survey, this case was well-known by all Dutch physicians, as a lot of media attention was given to it. However, at that moment a judicial decision was not yet made. Our findings quantitatively confirm those from a Dutch interview study from 2015, in which many physicians considered euthanasia in PWAD problematic, both legally and personally [17]. In that interview study, physicians were reluctant to forgo adequate verbal communication with the patient, because they wished to verify the voluntariness of a patient’s request, and the unbearableness of the actual suffering, and thus consider an AED of limited value in PWAD.

The Dutch law does not give restrictions towards euthanasia in PWAD if it is performed according to the obligatory due care criteria. We found in our study that the majority of GPs (54%) judged the acting of the doctor in the vignette case as not right. Recently, the supreme court in The Hague gave clearance and no punishment in the case on which our case vignette was inspired [20]. The argumentation of the Court is mainly based on the concept of ‘precedent autonomy’ and ‘continuity of person’, whereby the self-determination to be respected was made at the time the person was still competent. This argumentation is in line with the views of philosopher of law Ronald Dworkin. Dworkin emphasizes that critical interests –as coded in the AED- should take precedence over experiential interests (from flimsy games to poetry) as expressed by or observed from the patient [25]. According to legal philosopher Rozemond, this shows an overestimation of rational faculties of the human mind [26]. Rozemond doubts whether the previous competent self legally can prevail over the present, more or less incompetent self, commonly known as the “then-self-versus now-self” problem. In his reasoning it is a misconception that a loss of memory in dementia necessarily results in a diminished sense of self [27]. A more balanced perspective is suggested in which we factor in both the previously expressed wishes (respecting autonomy) and the current reality (respecting beneficence) [28].

In our study we found that two out of five GPs think that an AED cannot replace an oral request if communication with the concerned patient is impossible. Only one out of five think that it can replace it. As an AED now legally can replace an actual, oral euthanasia confirmation, a PWAD may not have the opportunity anymore to decide on his euthanasia request. Due to the progressive cognitive impairment, emotional – and/or behavioral problems in PWAD, regularly and carefully discussing and updating the person’s wishes becomes extremely import. Specifically because consistency in choice and regular conformation of consistency is legally mandatory. An earlier study also showed that many GPs would like to have training to increase their knowledge around AEDs [12]. Up to now, the Royal Dutch Medical Association (KNMG) guideline for physicians gives no requirements for an AED, but is currently developing a new guideline [29]. Consequently, at the moment it is uncertain whether an AED should be considered and thus made as a personal, conversational document, or that it should be a legal notarized pre-printed document, standardized according to the jurisprudence of the supreme court. When a PWD wants to discuss an AED, we recommend that GPs will take advantage of the opportunity, to also embark on the overarching advance care planning. By using the opportunity to provide realistic information about the dementia trajectory and its consequences, unrealistic fear for future suffering and loss of control may be relieved [30–32].

Although the Supreme Court ruling has confirmed the interpretation of the law, our study shows that GPs are very divided in their moral judgment regarding this law interpretation. The recent concerns of the United Nations- Human rights committee towards the Dutch euthanasia practice can be regarded as an opportunity to elaborate on the experiences of burden and the ethical dilemmas which GPs in the Netherlands are facing [33]. For example GPs could be supported by having opportunities for early ethical review. In line with this, an ethicist and former member of one of the regional euthanasia review committees recently publicly stated that in complex cases, like euthanasia in PWADs is, ethical and moral reflection is largely lacking [34]. She called for a “more proactive review” that is “being broader in scope” for complex cases by using a multidisciplinary approach [35]. This implies that there is also a need for a proactive, broader, multidisciplinary decision-making process for complex euthanasia requests of PWADs, for instance supported by moral case deliberation (MCD). A recent study showed that addressing harm in MCD aids healthcare professionals in the task they are facing [36]. Giving GPs better access towards MCD would be a sensible policy as it may counteract polarization within the profession and society. In our earlier study we also argued for creating awareness of the possibility to consult a spiritual care provider [13, 37]. These suggestions to introduce ethical review ex ante medical decisions on request for termination of life should be further explored, in order to address GPs’ personal moral considerations and dealing with social pressure. Better support for physicians in an early phase may be beneficial in all countries dealing with the legalization of euthanasia.

### Strengths and limitations

This quantitative survey is unique in focusing on Dutch GPs’ views on euthanasia requests for PWAD. The Dutch jurisdiction on euthanasia and PWAD is interestingly unique, but limited to Dutch territory. The questions raised ethically reach much further. We had a high response rate from all regions of the Netherlands. The relatively high response rate emphasizes GPs’ high involvement in this topic, as other surveys among Dutch GPs mostly had much lower response rates (around 30%) [38–40]. The respondents are representative for the Dutch GP practice, as checked for age, gender and region [36].

A limitation is that the survey was no validated questionnaire. However, the basic questionnaire relied on two previous studies and a literature review, and was adapted after having received feedback from six experts.

## Conclusion

In our exploration of the views of general practitioners confronted with an advance euthanasia request from a person with advanced dementia, the vast majority of the responding GPs experienced difficulties adequately judging the obligatory care criteria of the patients’ unbearable suffering with no prospective improvement. Even though euthanasia on the basis of a previous AED is now possible by law for PWAD in the Netherlands, only a minority of GPs support this. Therefore, it can be emphasized that research and development of moral and ethical support throughout the decision making process is needed.

## Supplementary Information

**Additional file 1.**

## Data Availability

The datasets used and/or analysed during the current study are available from the corresponding author on reasonable request.

## Abbreviations

AED: Advance euthanasia directive
GP: General Practitioner
KNMG: Royal Dutch Medical Association
MCD: Moral Case Deliberation
PW(A)D: Person with (Advanced) Dementia
PWDs: Persons with Dementia
SCEN: Support and Consultation on Euthanasia in the Netherlands
