# Supplementary material for: Euthanasia in advanced dementia; the view of the general practitioners in the Netherlands on a vignette case along the juridical and ethical dispute
Source: BMC Fam Pract. 2021 Nov 18;22:232. doi: 10.1186/s12875-021-01580-z (PMC8600859; doi:10.1186/s12875-021-01580-z)
Supplement: Supplementary file 1 — Additional file 1. [file 12875_2021_1580_MOESM1_ESM.docx]

# Additional file 1

**General questions**

1. What is your age?

……. years

2. What is your gender?

Male

Female


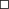


3. How many years of experience do you have as a general practitioner (GP)?

……. years

4. Do you work as a locum GP or as a regular GP?

Locum general practitioner

Regular general practitioner

5. Where do you work most of the time (4 numbers of Postal Code)?

6. How many advance euthanasia directives (AED) do you think you receive monthly?

<1 per month

1 per month

>1 per month

7. How much time do you spend on average discussing AEDs monthly?

<20 minutes per month

20 minutes per month

>20 minutes per month

-
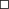


8. How often is, estimated, a euthanasia request for dementia included in the AED?

Less than 50%

About 50%

More than 50%

9. Have you ever contacted any of these healthcare professionals to discuss a case of a person with dementia? *(Several answers possible)*

Colleague GP


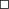


Elderly care physician


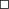


SCEN physician


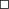


Consultant palliative care


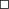


Health care chaplain

Humanistic caregiver


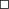


Spiritual care provider

Practice nurse specialized in psychiatrics

Psychologist

None

Other

10. Have you ever had a euthanasia **request** from a patient WITHOUT dementia?
 Yes

No 🡪 Skip to question 13

11. Have you ever **performed** euthanasia on a patient WITHOUT dementia?
 Yes

No

12. What kind of burden have you experienced regarding the euthanasia **request** and possible euthanasia **procedure**? *(Several answers possible)*

Pressure from the patient

Pressure from relatives

Emotional burden
 Uncertainty concerning the technical execution

Uncertainty concerning the mental competence

Uncertainty concerning the advance euthanasia directive

Time pressure


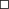


No burden
 Other

**Questions regarding to patients with dementia**

13. Have you ever had a euthanasia **request** from a patient WITH dementia?

*(Several answers possible)*

Yes, the patient was competent at that time

Yes, the patient was incompetent at that time

No 🡪 Skip to question 17

14. Have you ever **performed** euthanasia on a patient WITH dementia? (*Several answers possible)*

Yes, the patient was competent at that time

Yes, the patient was incompetent at that time

No

15. What form of burden have you experienced regarding the euthanasia **request** and possible euthanasia **procedure**? *(Several answers possible)*

Pressure from the patient

Pressure from relatives

Emotional burden
 Uncertainty concerning the technical execution

Uncertainty concerning the mental competence

Uncertainty concerning the advance euthanasia directive

Time pressure


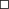


No burden
 Other

16. Please give a short outline of the situation in which there was ambiguity regarding the competency of the patient, if applicable.

17. Is it possible that you will perform euthanasia in a patient with dementia in the future?

Yes, I think that can be possible

No, but I will always refer these patients to a colleague

No, and I will never refer these patients to a colleague

18. Did the recent debates about euthanasia in patients with dementia influence your way of acting? *(Several answers possible)*

Yes, I am more reserved in performing euthanasia

Yes, I am more fearful for the legal processes

Yes, I am more prone to forward these patients to a colleague/end-of-life clinic


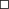


Yes, I consult other healthcare professionals more often

No, no influence

Other

19. Which treatment options regarding end of life listed below, do you discuss with your patients with dementia? *(Several answers possible)*

No antibiotics for infections

No life-extending treatments

No CPR

No hospital admissions

Palliative sedation

Physician-assisted suicide

Not applicable

**Support of healthcare professionals regarding euthanasia requests in patients with dementia**

If you have never had a euthanasia request of a patient with dementia, you can skip to question 21.

20. What kinds of support have you used when dealing with previous euthanasia requests or procedures? *(Several answers possible)*

Consulting palliative care

Geriatric consult team

Humanistic caregiver

End-of-life clinic (physician/nurse)

Moral deliberation


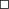


PaTz-group^1^**^^[[1]](#footnote-1)^^**

SCEN physician

Spiritual care provider

Other

21. What kinds of support would you like to use when dealing with euthanasia requests in the future? *(Several answers possible)*

Consulting palliative care

Geriatric consult team

Humanistic caregiver

End-of-life clinic (physician/nurse)

Moral deliberation


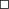


PaTz-group**^1^**

SCEN physician

Spiritual care provider

Other

22. How important do you rate the support of healthcare professionals in the different phases of the euthanasia procedure below on a scale of 0 to 10? (0 = not important; 10 = very important)

**Euthanasia request before starting the euthanasia procedure**

0 1 2 3 4 5 6 7 8 9 10

**During the euthanasia procedure towards the euthanasia performance**

0 1 2 3 4 5 6 7 8 9 10

**The performance of the euthanasia**

0 1 2 3 4 5 6 7 8 9 10

**The procedure after the performance of the euthanasia**

0 1 2 3 4 5 6 7 8 9 10

**Training regarding euthanasia in patients with dementia**

23. In which area would you like to increase your knowledge during training?

*(Several answers possible)*

Communication techniques to discuss end of life

Signaling symptoms (pain for example) in patients with a cognitive impairment


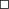


Dealing with the pressure by relatives


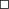


Legislation and interpretation of euthanasia regarding PWD

Advance care planning

Disease trajectory of dementia

How to compose and handle an advance euthanasia directive

No wishes to increase knowledge of skills

Other

**Due care criteria**

24. Meeting and judgment of the due care criteria are often considered to be complicated in euthanasia in patients with dementia. An example of this is the judgment of the patient’s unbearable suffering with no prospect of improvement. In what extent do you agree with the statements below?

a. I experience difficulties by judging the due care criteria of the patients’ unbearable suffering with no prospective improvement adequately in an incompetent patient.

Totally disagree Disagree Neutral Agree Totally agree

b. I am convinced that the due care criteria are met, when a competent patient considers his perspective of future suffering as unbearable, with no prospect of improvement.

Totally disagree Disagree Neutral Agree Totally agree

**Case vignette**
*The following questions are about a fictional situation that could occur during the last phase of life. Please, choose the answers that resemble your opinion the best.*

Mr. Smit is 70 years old and is indisputably diagnosed with dementia by a geriatrician. He does not recognize his wife and children anymore, refuses to eat, and increasingly isolates himself. Discussing his treatment is not possible anymore. Ten months ago, still being competent, he composed an advance euthanasia directive (AED), in which he declared that he would opt for euthanasia when suffering from dementia.

His family is now asking for performing this, given the patient’s AED and his unbearable suffering with no prospect of improvement. The general practitioner considers the patient incompetent, can imagine that the patient is unbearably suffering and is convinced that the patient’s AED can replace an oral request. The consulted SCEN physician^1^ and elderly care physician confirmed this and approved euthanasia. A sedative was orally administered to prevent possible unpredictable behavior, agitation and startle reactions at which the patient might walk away, after which the GP performed the euthanasia. After having received the written report of the euthanasia procedure from the GP, the regional review committee invites him to give an explanation of his actions.

*^1^SCEN; support and consultation on euthanasia in the Netherlands. SCEN physicians are available for support, information and formal consultation around euthanasia*

25a. Do you judge the way of acting of this general practitioner to be correct?

Yes

No

**My personal view regarding to euthanasia in patients with dementia is:**

b. An advance euthanasia directive can replace an oral request if communication with the concerned patient is impossible.

Yes

No

Maybe, if

c. The family can initiate the start of a euthanasia procedure representing the interests of the concerned patient.

Yes

No

Maybe, if

d. A sedative medicine prior performing euthanasia to the concerned patient is allowed.

Yes

No

Maybe, if

**Your own input**

If you feel like something you want to share is missing in the questionnaire, please write it down in the box below.

*This is the end of this questionnaire. We want to thank you very much for participating. If you are interested in the results of this study, write down your email address in the box below.*

*Your email address will only be used for sharing the results.*

*You can return the completed questionnaire in the attached self-addressed envelope.*

1. ^1^ Group of general practitioners and district nurses that debate six times a year under the supervision of a palliative care consultant to identify palliative care early, to act proactively. [↑](#footnote-ref-1)
